# Haemodynamic changes during propofol induction in dogs: new findings and approach of monitoring

**DOI:** 10.1186/s12917-018-1608-8

**Published:** 2018-09-12

**Authors:** Andrea Cattai, Roberto Rabozzi, Heidi Ferasin, Maurizio Isola, Paolo Franci

**Affiliations:** 1 Department of Animal Medicine, Production and Health, Agripolis, Università degli Studi di Padova, Padua, Italy; 2 CVRS – Policlinico Veterinario Roma Sud, Rome, Italy; 3 Specialist Veterinary Cardiology Consultancy, Lymington Bottom, Four Marks, Hampshire, UK

**Keywords:** Propofol, Haemodynamic effects, Baroreflex, Anaesthesia, Transthoracic echocardiography, Dog

## Abstract

**Background:**

Propofol is one of the most widely used injectable anaesthetic agents in veterinary practice. Cardiovascular effects related to propofol use in dogs remain less well defined. The main objective of this study was to evaluate the haemodynamic changes during induction of general anaesthesia with propofol in healthy dogs, by a beat-to-beat continuous monitoring. All dogs were premedicated with intramuscular acepromazine (0.015 mg/kg) and methadone (0.15 mg/kg). Transthoracic echocardiography was used to measure the velocity time integral (VTI) of the left ventricular outflow tract. A syringe driver, programmed to deliver propofol 5 mg/kg over 30 s followed by a continuous infusion of 25 mg/kg/h, was used to induce and maintain anaesthesia. From the initiation of propofol administration, heart rate (HR) and mean invasive arterial blood pressure (MAP) were recorded every 5 s for 300 s, while aortic blood flow was continuously recorded and stored for 300 S*. maximum* cardiovascular depression was defined the lowest MAP (MAP_Tpeak) recorded during the monitored interval. VTI and VTI*HR were calculated at 0, 30, 90, 120, 150 and 300 s post administration of propofol, and at MAP_Tpeak. Haemodynamic effects of propofol in relation to plasma and biophase concentrations were also evaluated by pharmacokinetics simulation.

**Results:**

The median (range) HR was significantly higher (*p* = 0.006) at the moment of maximum hemodynamic depression (Tpeak) [105(70–148) bpm] compared with pre-induction values (T0) [65(50–120) bpm]. The median (range) MAP was significantly lower (*p* < 0.001) at Tpeak [61(51–69) mmHg] compared with T0 [88(72–97) mmHg]. The median (range) VTI and VTI*HR were similar at the two time points [11.9(8.1–17.3) vs 13,3(9,4-16,5) cm, and 1172(806–1554) vs 1002(630–1159) cm*bpm, respectively].

**Conclusions:**

Induction of anaesthesia with propofol causes a drop of arterial pressure in healthy dogs, however cardiac output is well maintained by compensatory chronotropic response. The magnitude of MAP_Tpeak may be strictly related with propofol plasma concentration.

## Background

Induction of anaesthesia in dogs can be accomplished by using propofol (2,6-di-isopropylphenol), one of the most widely used injectable anaesthetic agents in veterinary practice. Cardiovascular effects related to propofol use have been subjected to intense research, often using the dog as a research model and with conflicting results. Blood pressure (BP) decrease is a common finding when propofol is used to induce anaesthesia in dogs [1–7]. Several mechanisms have been suggested as possible contributory causes of cardiovascular effects of propofol at clinically relevant concentrations: decrease in left ventricular preload produced by direct venous vasodilatation [8, 9]; reduction in systemic vascular resistance by arterial relaxation, most likely due to inhibition of sympathetic nervous system activity [2, 10] and negative inotropic effect [11–13].

The heart rate response to propofol administration in dogs remains less well defined. After different doses of propofol administered intravenous (IV) in dogs, either a significant increase of heart rate (HR) [1, 2] or no significant chronotropic effects [3–5, 7] have been reported. Propofol has also shown conflicting results on attenuation of baroreflex function in other species, either demonstrating no effect or attenuation related to propofol blood concentration [14–18].

One of the difficulties in monitoring hemodynamic changes during induction of general anaesthesia is the ability to record the rapidly occurring cardiovascular variations in a subject which moves from being fully conscious to being anaesthetised. Beat-to-beat monitoring of the cardiac stroke volume is crucial to accomplish this task. In this respect, transthoracic echocardiography has some advantages over other methods of cardiovascular monitoring, being a non-invasive technique that allows quantitative assessment of left ventricular function. Cardiac ultrasound machines are also widely available in the veterinary field and, therefore, it does not normally incur further financial expenses. Its use to monitor haemodynamic changes during anaesthesia has been reported [19, 20]. Doppler echocardiography can be used as a method to monitor velocity time integral (VTI) variations of the aortic blood flow during and after administration of drugs in dogs [21], which is commonly used to evaluate stroke volume (SV) variation on the same subject [21–26]. Pulsed Doppler echocardiographic assessment of cardiac output (CO) is a validated non-invasive method in humans [27, 28].

The aim of the present study was to investigate the haemodynamic changes produced by propofol, administered intravenously over 30 s in healthy dogs, through the use of continuous monitoring of electrocardiograph (ECG), invasive BP and trans-aortic flow over 300 s. Haemodynamic effects of propofol in relation to plasma and biophase concentrations were also evaluated by pharmacokinetics simulation.

## Methods

The study was approved by the Ethic Committee of the University of Padua (Prot. N. 89,556).

### Animals

Dogs admitted to the Ospedale Veterinario Roma Sud for various scheduled procedures were eligible for enrolment in the study. All animals underwent a physical examination and blood test analysis. Dogs were excluded from the study if owner consent was not granted, if the subject was assigned to American Society of Anesthesiology (ASA) physical status classification more than I, if they were < 1 year of age, or had a temperament that precluded use of a standard anaesthetic technique.

### Study protocol

Dogs were premedicated with acepromazine 0.015 mg/kg (Fatro S.p.A., Ozzano dell’Emilia, Italy) and methadone 0.15 mg/kg (Dechra, Bladel, Netherlands) mixed in the same syringe and administered intramuscularly. After 40 min, two over-the-needle catheters were aseptically inserted, one into a cephalic vein and another one in a dorsal pedal artery. Cardiovascular and respiratory variables were monitored by a multiparameter monitor (Datex Ohmeda AS/3, GE Healthcare). Dogs were positioned in a right lateral recumbent position and continuous ECG monitoring and invasive BP monitoring was started. Hair was clipped over the xiphoid area. Transthoracic echocardiography was used to measure the VTI of the left ventricular outflow tract, with a phased array PA240 probe (Esaote MyLab 70 CV). Sweep speed during recordings was set to 240 Hz (180–300 Hz). Optimised subcostal standard view of the left ventricular inflow and outflow tracts was used for data acquisition. Two-dimensional cine loops and Doppler tracings were recorded and stored on the internal hard drive of the echocardiograph and off-line analysed. The system was set to store raw DICOM data.

In order to induce anaesthesia a 50 ml syringe loaded with propofol (Esteve, Milano, Italy) was mounted on a syringe driver (Graseby 3500, Smiths Medical, England), which was programmed to intravenously deliver 5 mg/kg over 30 s, followed by a continuous infusion of 25 mg/kg/h to induce and maintain anaesthesia. At 2 min from initiation of the infusion the dog was assessed the first time to determine if endotracheal intubation was possible. When weakened palpebral reflex, rostromedial rotation of the eyeball, reduction in jaw tone and lack of tongue withdrawal were obtained, endotracheal intubation was facilitated and the dog was connected to a circle breathing system for 100% oxygen delivery. If a dog became apnoeic (no spontaneous ventilation for 30 s) or hypercapnic, it was manually ventilated until it was able to spontaneously maintain normocapnia (35– 50 mmHg).

Trans-aortic flow was continuously recorded for 300 s, after the beginning of the infusion. Heart rate and systolic, diastolic and mean arterial blood pressure (MAP) were also recorded every 5 s for 300 s, using an electronic spread sheet throughout available via a commercial software (Monitor Software version 6.1, University of Hong Kong). Data were downloaded onto a laptop computer connected to the anaesthesia monitor by a serial port adaptor. Aortic velocity time integral was obtained from digital still images as a mean of 10 consecutive measurements, which were obtained during spontaneous breathing. Only high-quality images were used for data analysis. All measurements were made off-line by an investigator (RR). Maximum cardiovascular depression was defined the lowest MAP (MAP_Tpeak) recorded during the monitored interval (Tpeak). VTI and VTI*HR (minute distance) were calculated at 0, 30, 90, 120, 150 and 300 s post administration of propofol, and at Tpeak.

A pharmacokinetics (PK) simulation of the anaesthetic protocol used in this study was carried out, running on a personal computer the Computer Control Infusion Pump V.2.0E software, programmed with a validated PK model [29] and a specific K_e0_ [30]. During the simulation this software provided data of the predicted propofol plasma concentration, and the related estimated effect-site concentration every 5 s.

### Statistical analysis

Continuous variables were checked for normal distribution by visual inspection of the bar graph, frequency of distribution and performing the Shapiro–Wilk normality test. Variables that were normally distributed were reported as the mean and standard deviation, whereas non-normally distributed variables were expressed as the median (range). Friedman Test was used to analyse differences within subjects and Dunn’s post hoc test was applied. The significant level was set at 5%. According to a priori power analysis (Power = 80%, α = 0.05), based on previously reported data on HR and MAP variations in dogs treated with a similar anaesthesia protocol [7], ≥4 subjects needed to detect significant effects. Statistical analysis was performed using MedCalc Software version 12.6.1.0 and Dell Statistica Software version 13.1.

## Results

Eight mixed breed dogs (2 males and 6 females) were included in this study. Median age and median body weight were 54 months (18–120 months) and 17 kg (6–35 kg), respectively. The median intubation time after the start of the propofol infusion was 140 (130–180) seconds. Neither apnoea, nor other anaesthetic-related adverse effects were observed.

**Fig. 1:**
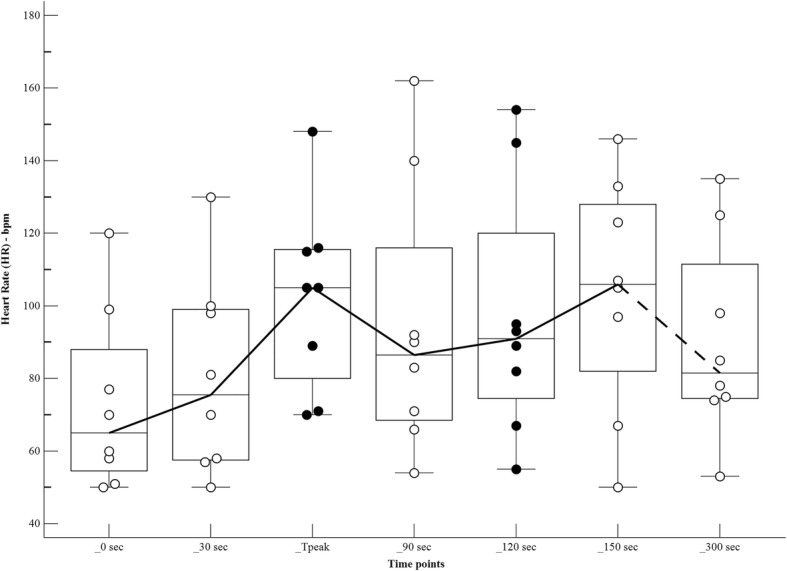
Values of heart rate (beats minute^− 1^) in dogs (*n* = 8) receiving a bolus of propofol at different time points. The central box represents the values from the lower to upper quartile. The middle line represents the median. Time points non-significantly different (empty circles) and significantly different (full circles) from the baseline

**Fig. 2:**
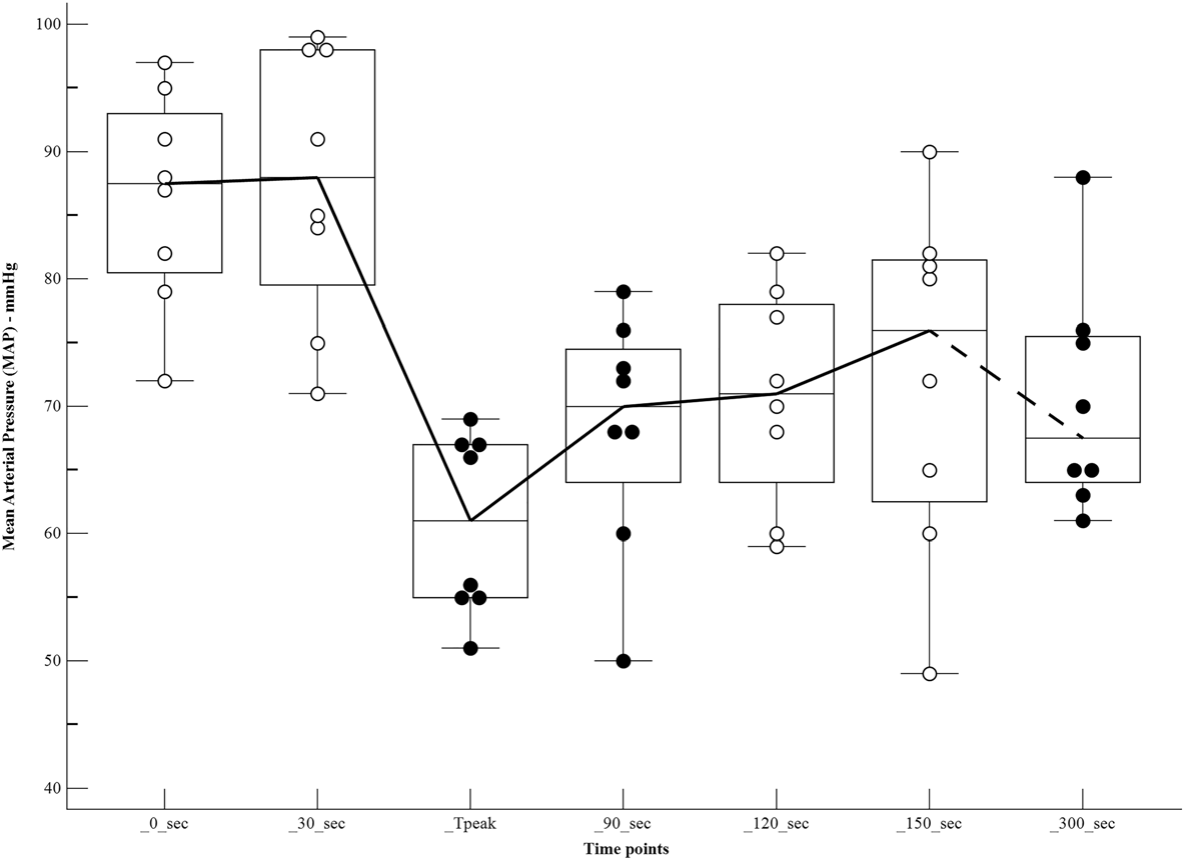
Values of mean arterial pressure (mmHg) in dogs (*n* = 8) receiving a bolus of propofol at different time points. The central box represents the values from the lower to upper quartile. The middle line represents the median. Time points non-significantly different (empty circles) and significantly different (full circles) from the baseline

**Fig. 3:**
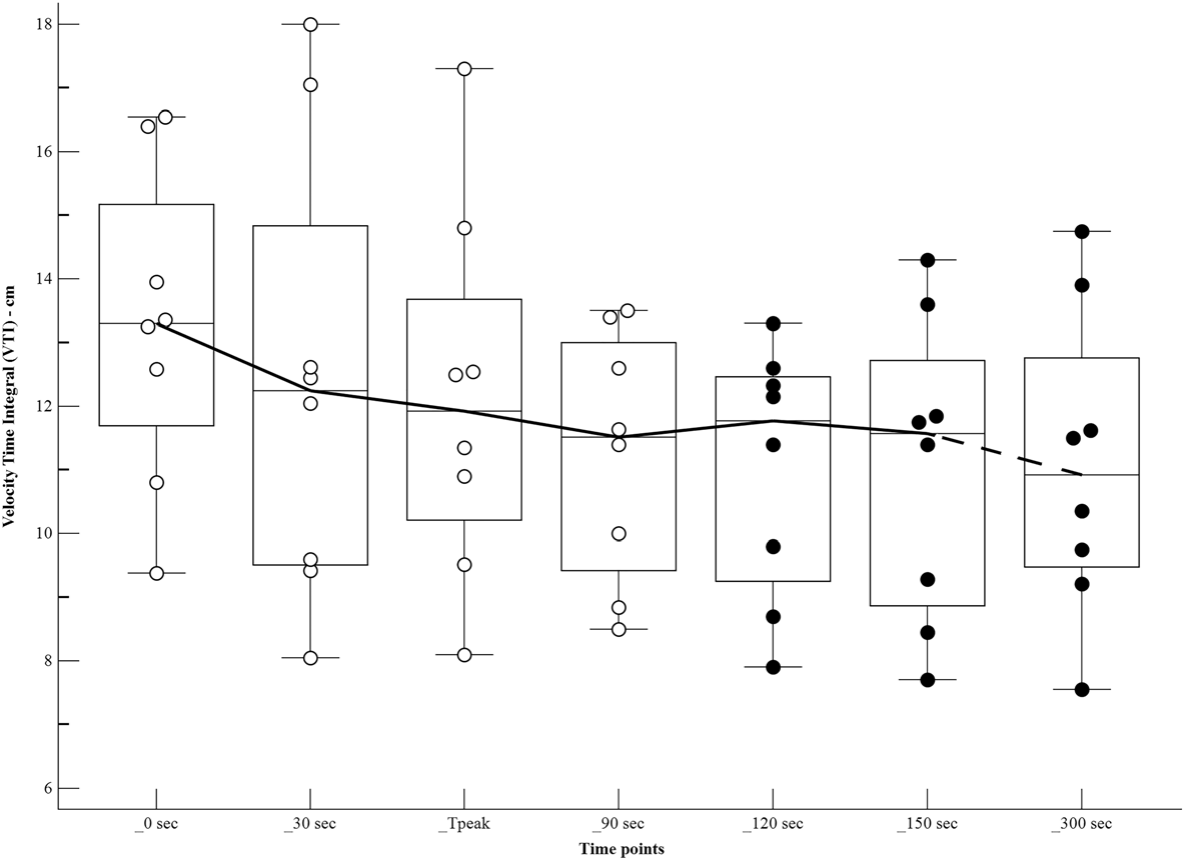
Values of velocity time integral (centimetres) in dogs (*n* = 8) receiving a bolus of propofol at different time points. The central box represents the values from the lower to upper quartile. The middle line represents the median. Time points non-significantly different (empty circles) and significantly different (full circles) from the baseline

**Fig. 4:**
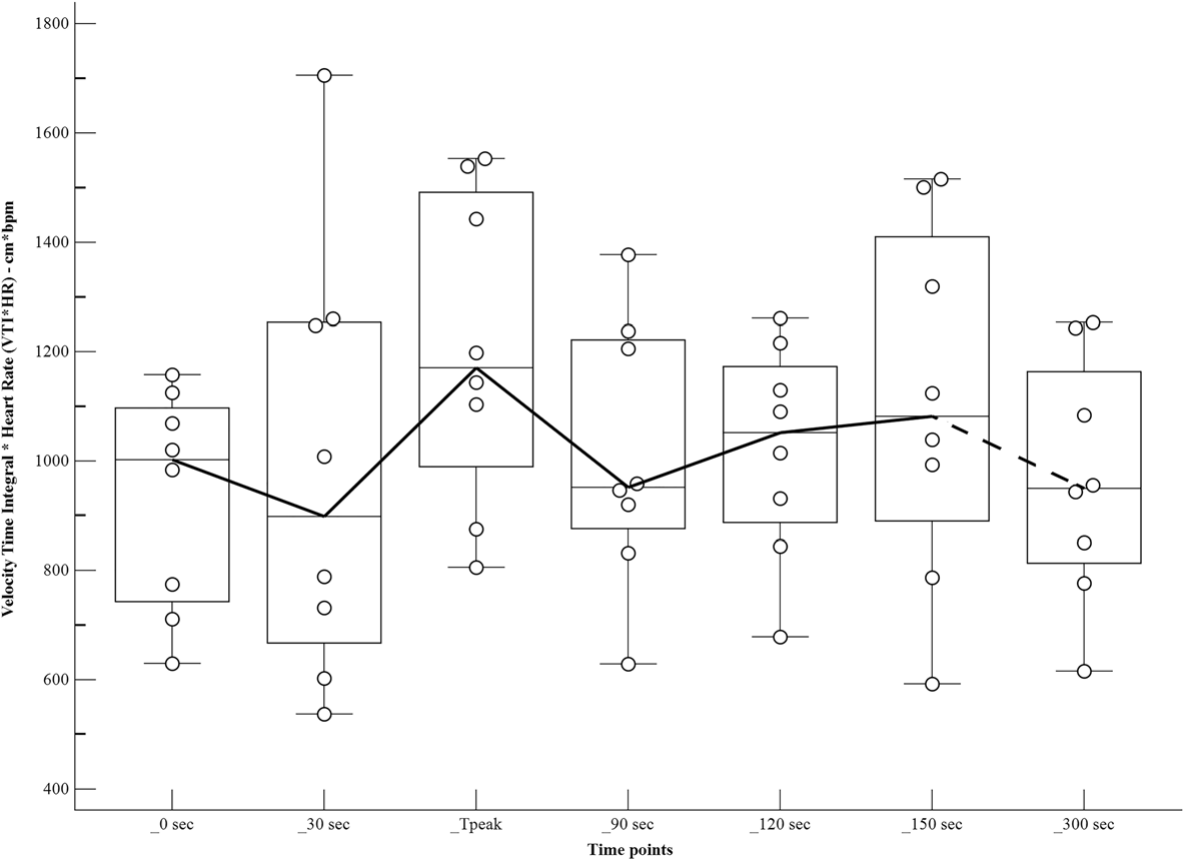
Values of velocity time integral*heart rate (centimetres*beats minute^− 1^) in dogs (*n* = 8) receiving a bolus of propofol at different time points. The central box represents the values from the lower to upper quartile. The middle line represents the median. Time points non-significantly different (empty circles) and significantly different (full circles) from the baseline

The changes in haemodynamic variables, at different time points after the bolus of propofol, are shown in Table 1 and Figs 1, 2, 3 and 4. There was a significant increase in the HR at Tpeak (*p* < 0.01), 120 and 150 s (*p* < 0.05), compared to the baseline. There was a significant decrease in the MAP at Tpeak (*p* < 0.001), 90 and 300 s (*p* < 0.05), and in the VTI at 120, 150 and 300 s (*p* < 0.05).

**Table 1:** Haemodynamic variables in dogs (*n* = 8) receiving a bolus of 5 mg/kg of propofol, administered over 30 s IV, at different time points

	Pre-induction	30 s	Tpeak 55 (50–60) sec	90 s	120 s	150 s	300 s
HR	65 (50–120)	76 (50–130)	105 (70–148))**	87 (54–162)	91 (55–154)*	106 (50-146)*	82 (53–135)
MAP	88 (72–97)	88 (71–99)	61 (51–69)***	70 (50-79)*	71 (59-82)	76 (49–90)	68 (61–88)*
VTI	13.3 (9.4–16.5)	12.2 (8–18)	11.9 (8.1–17.3)	11.5 (8.5–13.5)	11.8 (7.9–13.3)*	11.6 (7.7–14.3)*	10.9 (7.5–14.7)*
VTI*HR	1002 (630-1159)	899 (537–1705)	1172 (806–1554)	952 (629–1377)	1053 (678–1262)	1082 (593–1516)	950 (616–1254)

Tpeak, moment of maximum haemodynamic depression; HR, heart rate (beats minute-1); MAP, mean arterial pressure (mmHg); VTI, velocity time integral (centimetres)

Data expressed as median (range)

Values significantly different from pre-induction

*0.01 < *p* < 0.05

**0.001 < *p* < 0.01

***p < 0.001

Based on the pharmacokinetics simulation, the estimated peak plasma concentration (6.24μgml^− 1^) of propofol (Cmax) was reached after 30 s and at the time of equilibration between plasma and biophase (five and a half minutes) was 5.68μgml^− 1^.

## Discussion

In this study a bolus of 5 mg/kg of propofol, administered over 30 s IV, caused a rapid drop in arterial BP, however cardiac output was well maintained by a significant rise in HR.

The reduction in arterial BP, following propofol administration, can be due to any mechanism acting on both cardiac output and systemic vascular resistance. CO is expressed as the product of SV and HR. SV depends on three primary factors (preload, afterload, and myocardial contractility), all of which are interrelated. The direct negative inotropic effect of propofol seems to be relatively small when it is used within clinical concentrations [13, 31]. Most likely, the decrease in BP arises primarily as a result of the vasodilatory effect of propofol. This could be due to both, a reduction of sympathetic tone and a direct effect on smooth muscle. Induction of anaesthesia with propofol in dogs has been demonstrated to result in a dose-dependent decrease of systemic vascular resistance [2].

Arterial baroreflex function is an important short-term neural control system for maintaining cardiovascular stability. Attenuation of this reflex by propofol, due to a central or peripheral effect on sympathetic nervous activity, has been reported in humans [17, 18]. Sato et al. [18] reported that an estimated blood propofol concentration of 5 μg ml^− 1^ would result in baroreflex inhibition. Running the pharmacokinetics simulation of the protocol of administration used in our study, this concentration was already achieved and exceeded at the end of the 30 s bolus of propofol. Other studies, in humans and other species, have shown no or only slight effects on baroreflex sensitivity [14–16]. Our results in dogs seem to be in agreement with the latter. The continuous recording of arterial BP in this study allowed identification of the time of maximal hemodynamic depression as occurring after about one minute [55 (50–60) sec] from the start of propofol administration. Based on the pharmacokinetics simulation, the Cmax of propofol was reached close to MAP_Tpeak, while the peak of the estimated propofol biophase concentration occurred about four and a half minutes later. In humans, the maximal BP depression is reached about 2.5 min after the administration of a bolus of propofol to induce anaesthesia [16, 32]. This delay is similar to the time to peak propofol effect-site concentration that is reported for adult patients [32, 33]. This remarkable difference suggests that the mechanism mainly involved in causing the decrease of BP may be different between the two species. Looking at the time to peak effect in humans, which is quite close to the peak of cardiovascular depression, it is likely that a drop in BP in this species occurs mainly by a centrally mediated mechanism. Based on findings of our study in dogs the MAP_Tpeak happens much earlier than the peak effect after a bolus of propofol. This would suggest that haemodynamic depression may not be mainly mediated by a central mechanism but could rather be a peripheral interaction within the vascular bed.

The study setting presented in this work offers an acceptable solution when studying rapidly occurring hemodynamic events in a clinical environment. These studies have to accomplish with two substantial requirements, a continuous beat-to-beat monitoring, enable recording instantaneous variations of hemodynamic variables and an ethically acceptable way of doing it. Both transthoracic echocardiography and invasive blood pressure measurement could provide the above-mentioned tasks, allowing us to collect data before and over the entire induction time. Moreover, adequate equipment and clinical competence to run this type of cardiac scanning and images interpretation are commonly available in the vast majority of the veterinary hospitals.

A significant increase in heart rate after propofol administration was previously reported, but in chronically instrumented dogs [1, 2]. Other authors found a non-statistically-significant increase in heart rate after BP drop following induction with similar dose of propofol [4, 5, 7], however, cardiovascular variables were not measured immediately after injection of propofol and continuous monitoring methods were not used.

Without actual determinations of plasma propofol concentrations the predictions of the pharmacokinetics simulation could be imprecise, as related to the predictive performance of the Beths PK model (median bias of the predictions − 3.05%, median inaccuracy 27.15% in their validation study). In addition, the effect-site equilibration constant used to describe the delay equilibration between the plasma concentration and the drug effect, could not be the most accurate. The value of K_e0_ can be determined from complex studies combining blood concentrations with frequent measurements of drug effect, unfortunately, in the dog, it has been little studied.

Considering that the reduction in blood pressure following a bolus of propofol is closely related to blood plasma drug concentration, a different speed of injection of the bolus may well have produced different degrees of variation in blood pressure within this group of dogs, especially as this finding has already been demonstrated in sheep [34].

This study has some limitations. The use of acepromazine and methadone as a premedication before propofol induction may have had some impact on the cardiovascular system. Acepromazine is an α1-adrenergic receptor antagonist that induces dose-dependent negative haemodynamic changes in dogs: a low dose of 0.05 mg/kg (three times the dose used in this study) administered IV has been shown to potentiate isoflurane-induced vasodilation without altering CO [35]. However, the same dose administered intramuscularly was shown to reduce BP at 5 and 15 min and HR at 5 min, compared with baseline values [36]. Methadone alone could cause dose-dependent sedation and a decrease in HR [37], an effect most likely attributable to a centrally mediated increase in vagal tone [38]. Nevertheless, premedication facilitated arterial catheter placement, reduced the pre-operative stress and excitement in dogs which itself would have had a greater haemodynamic influence, due to sympathetic stimulation, and allowed to use properly the Beths PK model for propofol.

In this study the minute distance, used as analogue of CO, did not change significantly at MAP_Tpeak (mild increase). However, considering the fairly small number of subject, it is possible that a significant effect was not detected. Despite this limitation of the study, our finding is consistent with previous results in premedicated [7] or unpremedicated [2, 3] dogs, in which CO remained essentially unchanged from the pre-induction value after administration of a similar or higher dose of propofol (4 to 15 mg/kg). Nevertheless, neither SV nor CO was directly measured throughout this study. The CO can be calculated by multiplying the cross-sectional area of the aorta * VTI * HR, however, comparing different values on the same subject, the first can be omitted in the calculation. In order to measure SV variation, the left ventricular outflow tract (LVOT) VTI was monitored. LVOT-VTI, or ‘stroke distance’, is commonly used as SV surrogate to measure variation of the left ventricular ejection on the same subject in human clinical studies [24–27]. Its use is reported also in animals [21–23]. VTI*HR, or ‘minute distance’, is directly related to cardiac output and has often been used as a useful alternative measure to evaluate the CO, particularly when more invasive methods are not available [21, 27, 39–41].

In this study, neither apnoea, nor other anaesthetic-related adverse effects were observed during induction and in the following minutes, despite these reported to be possible after propofol administration in other studies [42, 43]. Observing HR trend at the different time points a second peak at 150 s can be notice. This could be a consequence of a sympathetic stimulation, due to endotracheal intubation [32].

## Conclusions

In conclusion, this study has found that in healthy premedicated dogs, a bolus of propofol administered IV over 30 s causes a transitory drop in blood pressure, however the cardiac output was well maintained due to a raise in heart rate. We have also found that MAP_Tpeak happens closely to predicted Cmax and, therefore, their magnitude may be strictly related. To investigate how other drugs commonly used as part of canine anaesthetic protocols (such as alpha2-agonists or short-acting opioids) can affect reflex heart rate response following propofol administration, and how a reduction of the magnitude of Cmax can mitigate MAP_Tpeak, may be an interesting field of research.

## Abbreviations

BP: Blood pressure
Cmax: Estimated peak plasma concentration of propofol
CO: Cardiac output
HR: Heart rate
IV: Intravenous
LVOT: Left ventricular outflow tract
MAP: Mean invasive arterial blood pressure
MAP_Tpeak: Lowest MAP recorded
PK: Pharmacokinetic
SV: Stroke volume
Tpeak: Moment of maximum hemodynamic depression
VTI: Velocity time integral
